# Variability of *PRDM9* in buffaloes

**DOI:** 10.3389/fgene.2024.1479287

**Published:** 2025-01-09

**Authors:** Luca Godoi Rocha Santana, Jackeline Santos Alves, Fabieli Loise Braga Feitosa, Victoria Camilla Parente Rocha, Humberto Tonhati, Raphael Bermal Costa, Gregório Miguel Ferreira de Camargo

**Affiliations:** ^1^ Escola de Medicina Veterinária e Zootecnia, Universidade Federal da Bahia (UFBA), Salvador, Bahia, Brazil; ^2^ Departamento de Zootecnia, Universidade Estadual Paulista (Unesp), Jaboticabal, São Paulo, Brazil

**Keywords:** SNP, bubalus bubalis, recombination, alleles, genetic variability

## Abstract

The buffalo population raised in Brazil tend to show loss of genetic variability over generations, with significant estimates of inbreeding depression. Besides mating genetically distant individuals, other tools can be used to maintain/increase the genetic variability of the population, such as the use of *PRDM9* genotypes. The *PRDM9* gene promotes the creation of crossing-over points across the genome, with each allele promoting the creation of a different hotspot. Thus, increasing the frequency of less frequent alleles in the population, allows the emergence of new haplotypes and increases genetic variability. So, this study aimed to characterize the alleles of the *PRDM9* gene circulating in the Murrah, Jaffarabadi, and Mediterranean breeds and verify their potential impact on genetic diversity management within the populations. The three alleles (B, C and D) were found in the three breeds at different frequencies, as well as the genotypic frequencies. The mating of different homozygous genotypes and genotypes carrying less frequent alleles may increase recombination rates and population variability. Four described variants and one new variant for allele D were found by sequencing. It was verified that it is possible to mate sires and dams with different *PRDM9* genotypes in order to try to increase genetic variability in buffalo populations, improving the matings choices in buffalo breeding, helping to maintain production levels.

## Introduction

Brazil has about 1.59 million heads of buffalo (FAO, 2024) with herds for milk, meat, and dual-purpose production under genetic evaluation (ABCB, 2024). Studies on the population structure of some breeds raised in the country have been conducted and reported inbreeding coefficients of 3.5% (Nascimento et al., 2021) for the Murrah breed, 2.02% for the Mediterranean breed (Malhado et al., 2013), and 4.22% for the Jaffarabadi breed (Ferraz et al., 2015). Although inbreeding coefficients are low, inbreeding depression for weight, milk production, and reproductive traits has been reported in the Murrah and Mediterranean breeds (Santana et al., 2011; Malhado et al., 2013). Nascimento et al. (2021) reported a genomic inbreeding coefficient (in 1 Mb homozygosity regions) of 7.2% for the Murrah breed, and these values are increasing over generations, suggesting monitoring of matings.

Maintaining genetic variability is essential for achieving gains through selection. Inbreeding measures intra-locus genetic variability, but there is the inter-loci genetic variability given by the recombination event. The recombination occurs at specific sites and is guided by the PRDM9 (PR/SET Domain 9) protein (Myers et al., 2010). Each *PRDM9* allele promotes a different protein that performs crossing-over in a particular chromosomal region (Neale, 2010). The PRDM9 is a three functional domain protein, with the terminal one being a zinc finger (ZF) of cysteine2 histidine2 (C2H2) type (Parvanov et al., 2010). The tandem array of C2H2 zinc fingers and the single amino-acid change within the zinc fingers are responsible for creating the alleles. Each *PRDM9* allele breaks the double stand in a specific spot and promotes the recombination at that site (Parvanov et al., 2010; Myers et al., 2010; Ahlawat et al., 2017). Therefore, increasing the frequency of less recurrent alleles of this gene in the population helps to increase/maintain inter-loci genetic variability (Gonen et al., 2017).

In ruminants livestock (sheep, goat, buffalo, yaks and cattle), some *PRDM9* alleles have been characterized (Ahlawat et al., 2016; Ahlawat et al., 2017). In cattle, a concrete involvement in recombination events was confirmed (Shen et al., 2018; Zhou et al., 2018), even affecting fertility (Li et al., 2020; Seroussi et al., 2019). Moreover, the gene was reported to be under selection (Wang et al., 2019) and potentially useful to manage diverse in inbred population (Rocha et al., 2023).

Ahlawat et al. (2017) characterized the alleles of the *PRDM9* gene in buffaloes, totaling 14 different alleles. The alleles of this gene are characterized by the number and type of zinc finger (ZF) domains they possess. Thus, the present study aimed to conduct a preliminary investigation of the alleles occurring in populations of three buffalo breeds (Murrah, Jaffarabadi, and Mediterranean) and their potential use for maintaining and promoting genetic variability.

## Materials and methods

The project was approved by the Animal Use Ethics Committee of the Escola de Medicina Veterinária e Zootecnia of the Universidade Federal da Bahia (81/2018). Hair follicles from of a total of 85 buffaloes (females and males) of Murrah (n = 27), Jaffarabadi (n = 41), and Mediterranean (n = 17) breeds from six commercial farms were collected. The farms were located at São Sebastião do Passé-BA, Nova Andradina-MS, Registro-SP, Tietê-SP, Sarapuí-SP and Paracuru-CE, all in Brazil) in order to be as diverse and representative as possible. The DNA extraction was done using the NucleoSpin^®^ Tissue DNA extraction kit (Macherey-Nagel). The initial number of samples per breed was 50 animals, but the amplification of this region is complicated since it is a high polymorphic region with ZF domain repetitions.

For the amplification of the DNA sequence corresponding to the *PRDM9* gene region, the primers (F: ACC​TAG​ATG​ATT​AGT​GGG​GCG and R: GCT​GCA​GTA​ATT​CTC​CTG​TGA​C) described by Ahlawat et al. (2017) were used. PCR reactions were carried out with a total volume of 25 µL containing: 50–110 ng of genomic DNA, 0.8 µmol of each primer, 9 µL of Promega Taq Mix, and 12.4 µL of deionized water. The PCR reaction was subjected to the Veriti 96 Well Thermal Cycler (Applied Biosystems) using the following conditions: an initial denaturation step at 95°C for 4 min, followed by 34 cycles of 95°C for 45°s, annealing at 60°C for 45°s, extension at 72°C for 45°s, and a final extension of 8 min at 72°C. The amplification products were verified on 2% agarose gel stained with Sybr Gold. The amplified fragments were visualized under ultraviolet light and photographed with the L-PIX Transilluminator Molecular Imaging (Loccus Biotecnologia). Allelic and genotypic frequencies and Hardy-Weinberg equilibrium were calculated using Excel software. Genotypic frequencies were compared across breeds, in pairs, by chi-squared (GraphPad -https://www.graphpad.com/quickcalcs/chisquared1/). The PCR products of homozygous individuals were purified with 20% polyethylene glycol and sequenced using the BigDye v3.1 sequencing kit (Applied Biosystems, Foster City, CA, United States) on a 3500xl Genetic Analyzer (Applied Biosystems) according to the manufacturer’s instructions, using both primers to determine the ZF domain sequences. The obtained sequences were analyzed in CodonCode Aligner software and later deposited in GenBank (PP830922-26).

## Results and discussion

**FIGURE 1:**
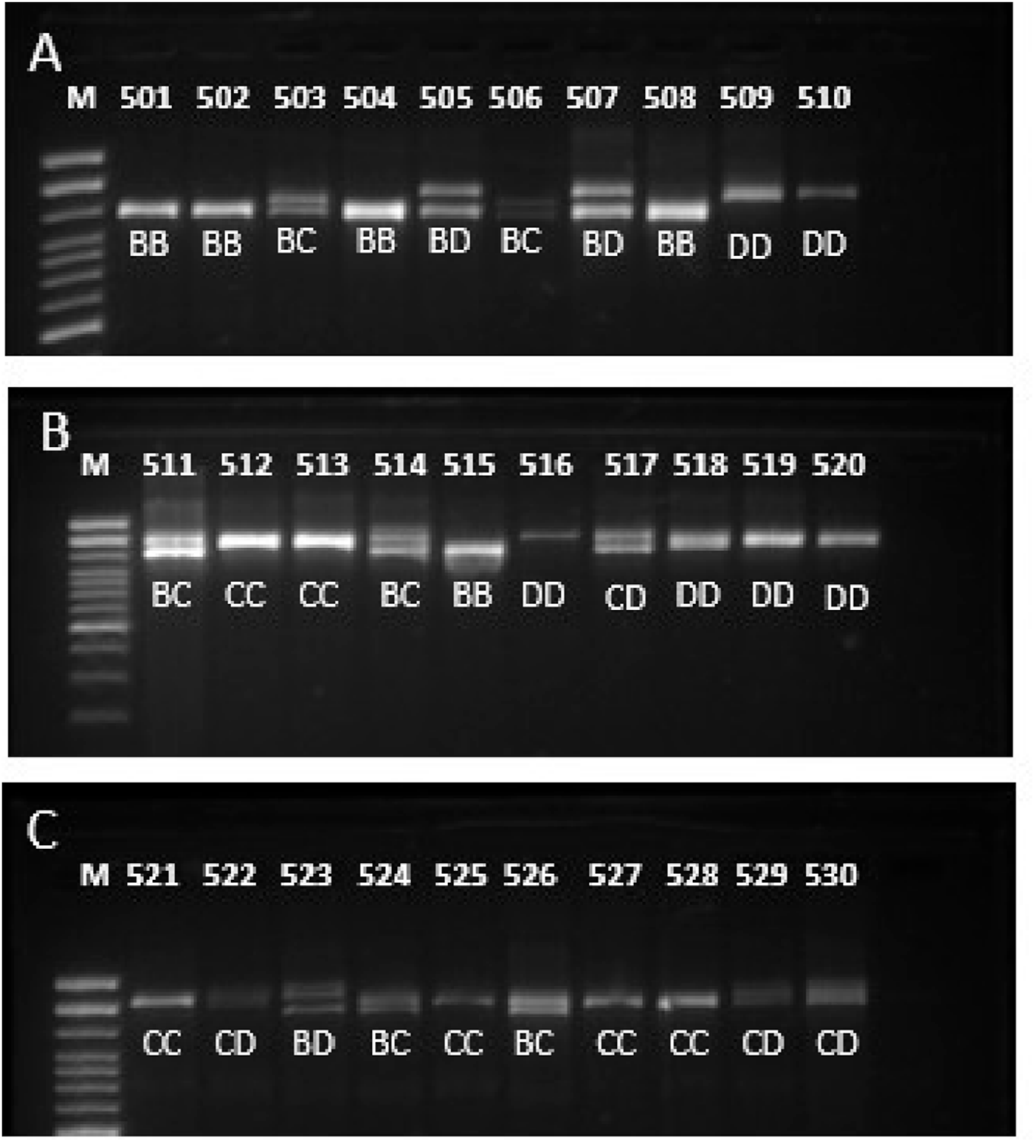
Gel image of PCR product of ZF domain of *PRDM9* in three buffalo breeds **(A)** Murrah, **(B)** Jaffarabadi and **(C)** Mediterranean showing some genotypes identified.

For the three studied breeds, the three described alleles B, C and D, and six genotypes were found, except for the CC genotype for the Murrah breed (Table 1; Figure 1). The samples were run twice in agarose gel to confirm the correct genotype. It was found that the Jaffarabadi population was out of Hardy-Weinberg equilibrium, while the others were in equilibrium (data not presented). After sequencing the homozygous, the buffalo 1 and buffalo 2 alleles (allele B), buffalo 3 (allele C), buffalo 12, and buffalo 15 (allele D) were observed.

**TABLE 1:** Allelic and genotypic frequencies for the *PRDM9* gene in buffaloes.

Breed	Alelle			Genotype					
	B	C	D	BB	BC	BD	CC	CD	DD
Murrah	0.48	0.07	0.45	0.19	0.11	0.48	0	0.03	0.19
Jaffarabadi	0.43	0.10	0.47	0.12	0.05	0.32	0.05	0.02	0.44
Mediterranean	0.27	0.41	0.32	0.06	0.23	0.18	0.18	0.23	0.12

The allelic and genotypic frequencies varied between breeds, as reported by Ahlawat et al. (2017), being these the first frequencies estimates of the *PRDM9* for the Mediterranean breed. The genotypic frequencies were significantly different across breeds (data not shown). The differences might be related to breed formation and selection strategies. The presented frequencies used a larger number of animals than Ahlawat et al. (2017), who used five to 12 animals for allelic characterization. For Jaffarabadi, the highest genotype (DD) and allele (D) frequencies are the same between studies, but all the six genotypes were herein reported while Ahlawat et al. (2017) reported three. For Murrah, the highest allele (B × D) and genotype (BD × CD) frequencies varied between studies. It might be due to sample size and population characteristics.

The distribution of *PRDM9* genotypes in the populations allows defining mating strategies to increase the frequency of some alleles, and genotypes in heterozygosity, aiming to increase the crossing-over rates and inter-loci variability. For Jaffarabadi breed, choosing to mate CC genotype animals with other homozygous tends to increase the frequency of allele C and heterozygous genotypes, promoting crossing-over in different regions and increasing the variety of haplotypes in the population. For Murrah breed, besides mating among homozygous, mating animals with allele C in heterozygosity in their genotype is also interesting for the same purposes. In the Mediterranean breed, genotypic frequencies are equally distributed, indicating a better distribution of recombination in different hotspots. A similar approach was already carried out in the Sindhi cattle (Rocha et al., 2023), which has low intrapopulation variability with good mating strategy options using *PRDM9* genotypes. The mating decisions may help to increase genetic variability and increase productivity as consequence.

The HW equilibrium calculation showed that the Jaffarabadi population is out of equilibrium. The higher frequency of homozygous in the observed frequencies compared to the expected ones is an indication of inbreeding, already evidenced in a previous study (Ferraz et al., 2015), being the breed with the highest inbreeding coefficient among those studied. It possibly happens because this breed has the smaller number of animals and natural service is the most common reproductive technique, being more frequent to mate related animals.

**FIGURE 2:**
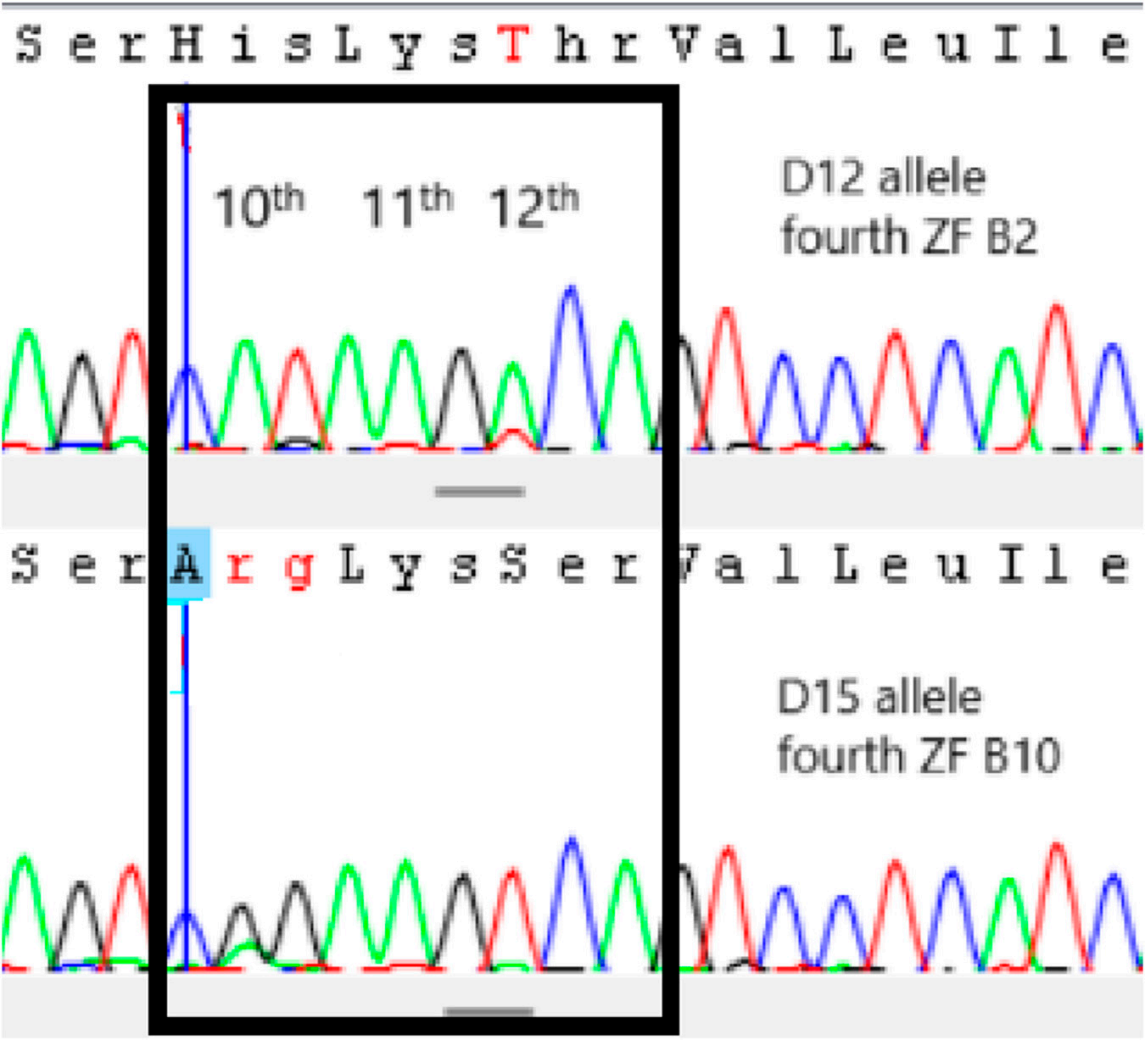
Sequence example of *PRDM9* polymorphisms. The black box indicates the codons of the 10th, 11th and 12th amino acids of fourth zinc finger (ZF) of allele D12 (ZF–B2) and D15 (ZF–B10). B2 and B10 ZFs differ from each other in 10th and 12th amino acids, changing a His (CAT) to an Arg (CGG) and a Thr (ACA) to a Ser (TCA), respectively.

The definition of *PRDM9* alleles is primarily given by the number of zinc finger domains. Allele B has seven ZF repetitions, allele C has eight repetitions, and allele D is characterized by nine ZF repetitions, initially characterized in agarose gel. However, the ZFs that constitute the alleles can vary, with the final characterization being done by sequencing, using numbers to identify them (buffalo 1-B1, buffalo 2-B2, etc.). All homozygous animals were sequenced, and four alleles (buffalo 1-B1, buffalo 2-B2, buffalo 3-C3, buffalo 12-D12) (Genbank: PP830922-25) out of the 14 described by Ahlawat et al. (2017) and a new one named buffalo 15-D15 (Genbank: PP8309226) were found. The ZF sequences of the new allele was B1-B9-B2-B10-B3-B3-B3-B5 following the initial description by Ahlawat et al. (2017). This allele differs from the others due to the unique sequence combination of ZFs not yet reported. It is important since it provides more options for recombination sites and strategic matings. Of the 41 homozygous animals identified in agarose gel (11 BB, five CC, and 25 DD), it was possible to identify the genotype of five Murrah (two B1B1, two B2B2, and one B1B2), seven Jaffarabadi (three B2B2, one D12D12, and two D15D15), and four Mediterranean (one B1B1, two C3C3, and one D12D12). A sequence example was given in Figure 2. It is important to notice that, for *PRDM9* gene and Sanger sequencing, it is possible to identify the alleles of animals with the same ZF number (BB, CC and DD) and with the same sequence (B1B1, B2B2, C3C3, D12D12 and D15D15) or with slight identifiable codon changes (B1B2). It happens because this gene is extremely polymorphic and, with more than one SNP, it is impossible to define the linkage phase to establish which allele it is. This is an interesting result because there is a large number of heterozygous in the populations concerning the ZF sequences, which is related to higher recombination rates.

*PRDM9* genotypes are another tool for maintaining variability that should be used to support other tools. It is important to remember that the way to increase intra-loci variability include mating non-related animals to try introduce new alleles. The methodology presented here can help maintain variability by increasing recombination rates, that is, circulating haplotypes, contributing to inter-loci variability with the possibility of increasing genetic gains (Gonen et al., 2017). Future studies with a larger number of animals and application of next-generation sequencing technology (for precise heterozygous identification) would permit a better scenario mating strategy. Moreover, the effectiveness of mating strategies in increasing variability over generations is a trial that should be planned and was not conducted yet.

## Conclusion

Several circulating alleles of the *PRDM9* gene were identified in the three studied buffalo breeds, indicating that they can help as an additional tool in increasing/maintaining genetic variability by choosing sire and dams with different *PRDM9* genotypes. It is also a strategy possible to be applied in any other livestock whose conditions were similar.

## Data Availability

The data presented in the study are deposited in the Genbank website repository, with the accession numbers PP830922-26.
